# Everolimus-induced effector mechanism in macrophages and survivability of Erdman, CDC1551 and HN878 strains of *Mycobacterium tuberculosis* infection

**DOI:** 10.1515/bmc-2021-0006

**Published:** 2021-06-01

**Authors:** Ruoqiong Cao, Kimberly To, Nala Kachour, Abrianna Beever, James Owens, Airani Sathananthan, Pooja Singh, Afsal Kolloli, Selvakumar Subbian, Vishwanath Venketaraman

**Affiliations:** College of Osteopathic Medicine of the Pacific, Western University of Health Sciences, Pomona, CA, USA; College of Osteopathic Medicine of the Pacific, Western University of Health Sciences, Pomona, CA, USA; Graduate College of Biomedical Sciences, Western University of Health Sciences, Pomona, CA, USA; Graduate College of Biomedical Sciences, Western University of Health Sciences, Pomona, CA, USA; College of Osteopathic Medicine of the Pacific, Western University of Health Sciences, Pomona, CA, USA; College of Osteopathic Medicine of the Pacific, Western University of Health Sciences, Pomona, CA, USA; Department of Pulmonary, Allergy, and Critical Care Medicine, The University of Alabama at Birmingham, Birmingham, AL35294, USA; The Public Health Research Institute at New Jersey Medical School, Rutgers University, Newark, NJ 07103, USA; The Public Health Research Institute at New Jersey Medical School, Rutgers University, Newark, NJ 07103, USA; The Public Health Research Institute at New Jersey Medical School, Rutgers University, Newark, NJ 07103, USA; College of Osteopathic Medicine of the Pacific, Western University of Health Sciences, Pomona, CA, USA; Graduate College of Biomedical Sciences, Western University of Health Sciences, Pomona, CA, USA

**Keywords:** Mycobacterium tuberculosis, Everolimus, foamy macrophages

## Abstract

With a disease as widespread and destructive as tuberculosis, more effective drugs and healthcare strategies, in addition to the current antibiotics regimen, are crucial for the enhanced well-being of millions of people suffering from the disease. Host-directed therapy is a new and emerging concept in treating chronic infectious diseases, such as tuberculosis. Repurposing of anti-cancer drugs, such as everolimus, may be an effective way to supplement the standard antibiotic treatment. Individuals with type 2 diabetes are increasingly susceptible to co-morbidities and co-infections including *Mycobacterium tuberculosis*, the causative agent of tuberculosis. We demonstrated in this study that *in vitro* everolimus treatment of granulomas from individuals with type 2 diabetes caused significant reduction in the viability of *Mycobacterium tuberculosis*.

Further investigations revealed the effects of everolimus in targeting foamy macrophages, a macrophage phenotype that forms around granulomas, and is characterized by a higher lipid accumulation inside the cells. These foamy macrophages are thought to harbor dormant bacilli, which are potential sources of disease reactivation. Therefore, blocking foamy macrophage formation would help better killing of intracellular bacteria. Here, we report the potential of everolimus treatment to downregulate lipid content within the foamy macrophages of *in vitro* granulomas, thus leading to a potential decrease in the number of foamy macrophages and a more robust response to *Mycobacterium tuberculosis*.

## Introduction

Tuberculosis (TB), an infectious disease caused by *Mycobacterium tuberculosis* (*M. tb*), is estimated to infect nearly 10 million people a year [1]. *M. tb* is transmitted through inhalation of aerosolized droplets, which first infects the alveoli in the lungs and then spreads throughout the body, infecting other organs. To control the infection, macrophages ingest and destroy the *M. tb* pathogen. While some of the infection may be cleared with bacterial killing, the macrophages begin to aggregate around the infected cell and create a granuloma, which acts to enclose and suppress the infection instead of clearing it, leading to latent tuberculosis (LTBI). Currently, the infection remains latent in about 1.7 billion people around the world [1]. The granuloma suppresses *M. tb* replication by creating a hypoxic environment, depriving the pathogen of the oxygen and nutrients it needs to replicate. Thus, the infection becomes localized within the lungs and can no longer be transmitted to other organs [2,3]. However, LTBI cases can reactivate to symptomatic TB disease upon host-immunocompromising conditions. Thus, immunocompromised individuals are more susceptible to reactivation of LTBI [4]. Specifically, individuals with type 2 diabetes mellitus (T2DM) are at a greater risk for developing active TB, likely due to alterations in immunity due to elevated blood glucose levels. It has been shown that individuals with T2DM have increased proinflammatory cytokines, increased oxidative stress, decreased levels of cytokines involved in controlling *M. tb* infection, and decreased levels of glutathione (GSH) in macrophages, which can hinder the formation of granulomas and the control of *M. tb* infection [5].

The current treatment for TB, namely the directly observed treatment short-course (DOTS), consists of a variety of antibiotics, including isoniazid (INH), rifampicin (RIF), pyrazinamide (PZA), and ethambutol (ETH), administered during the two-month initial phase, followed by INH and RIF in the continuation phase for four months. However, this treatment has higher rates of non-compliance due to side effects of the drugs, prolonged duration of therapy, and unawareness of patients. This non-compliance can lead to the development of drug-resistant TB, which is more lethal and very difficult to treat [6]. Since it is more likely for immunocompromised individuals to develop an active TB infection, it is crucial to create therapies that will be more successful than the current options, especially with the threat of developing resistant strains of *M. tb*. More recently, a promising research has been conducted to treat TB with host-directed therapy (HDT), which targets boosting the host immune functions to fight infection. HDT is different from antibiotics because it modulates the host cell functions; the drug everolimus, for example, can induce autophagy in host cells, which is capable of inhibiting intracellular *M. tb* growth [7,8].

Everolimus is an inhibitor of the mammalian target of rapamycin (mTOR) serine/threonine kinase signal transduction pathway. mTOR regulates multiple cellular processes involved in cell growth and differentiation; it is involved in metabolic processes, such as lipid metabolism, glucose metabolism, and protein metabolism, and inhibiting catabolic processes such as autophagy [7,9]. Autophagy is a crucial cellular defense mechanism in which damaged elements are removed by the cell via lysosomal degradation. This natural mechanism has been targeted to be used as a possible therapeutic method to rid cells of intracellular pathogens such as *M. tb* [10]. Since mTOR activity is increased in cells infected with *M. tb* [11], autophagy may be inhibited more than normal. Everolimus may be able to reverse these effects by inhibiting mTOR activity. Although everolimus is known to be an immunosuppressant used for patients with organ transplants, it has been suggested that low doses of everolimus improve immune response [12]. Everolimus has been used as a treatment for certain cancers and shows promising effects as a treatment for *M. tb*. This is due to its high water solubility and relatively low intracellular half-life [13]. Recently we reported that everolimus enhanced autophagy and restricted *M. tb* growth within an *in vitro* human granuloma when treated with a combination of first-line anti-TB drugs [8]. We also found that everolimus was in favor of modulating inflammation levels, oxidative stress, and the cytokine profile of granulomas during *M. tb* infection [8].

In this study, we determined the effects of everolimus treatment in improving the ability of *in vitro* granulomas generated from immune cells isolated from individuals with uncontrolled diabetes to control of *M. tb* infection. Our study findings reveal that everolimus treatment significantly diminished the viability of *M. tb* in the in vitro granulomas from individuals with T2DM. To understand the underlying mechanisms behind these effects, additional preclinical studies were performed in THP-1 cells.

*M. tb* infection is known to have characteristic effects on lipid metabolism [14]. Such changes in lipid metabolism are responsible for the development of foamy macrophages. Also known as foam cells, these are a type of macrophage that is packed with cholesterol and other lipids in the form of lipid bodies and are known to create fatty deposits on blood vessel walls. During *M. tb* infection, the granulomas become highly vascularized and recruit lymphocytes, macrophages, and dendritic cells to fight the infection. As the granuloma matures and macrophages differentiate, foamy macrophages begin to develop due to an uneven amount of low-density lipoprotein (LDL) entering and exiting the cell [15]. These foam cells have been shown to increase inflammation [16], and can keep *M. tb* infection in a vegetative, non-replicating state, creating a nutrient-rich reservoir favorable for the bacteria’s persistence. However, the foam cells become unable to ingest, kill, or otherwise respond against the bacteria [17]. Frédéric Altare’s team demonstrated that foamy macrophages could not mediate phagocytosis of new bacteria and showed decreased antimycobacterial capabilities [18]. In their study, Peyron et al. also showed that *M. tb* survived and remained in a dormant state inside foamy macrophages, which can provide the mycobacteria with nutrients and form a secure environment [18]. Therefore, decreasing the levels of lipid components of the foamy macrophages could be a potential way to control the viability of *M. tb* within these cells. Our current report builds upon our past studies on the effects of everolimus on *M. tb* infection of peripheral blood mononuclear cells (PBMCs) isolated from T2DM patients. Specifically, we evaluated the effects of everolimus in altering the levels of lipid bodies within macrophages infected with clinical *M. tb*. Strains, CDC1551 and HN878. We hypothesize that treatment with everolimus may play a role in decreasing the amount of lipid bodies within the *M. tb* infected macrophages, allowing these cells to control *M. tb* infection more effectively.

## Materials and Methods

### T2DM Subjects Recruitment and Human Blood Collection

Eight individuals with T2DM between the ages of 18 to 65 years old were recruited for this study. The T2DM individuals had no history of HIV infection or TB, and they had a glycated hemoglobin (HbA1c) level of more than 8.0%. Exclusion criteria for the T2DM subjects also included hepatitis, pregnancy, and medication history of metformin, which plays a role in *M. tb* inhibition [19,20]. After obtaining written informed consent, 40 mL of whole blood was drawn from the patients and collected in acid citrate dextrose tubes by a licensed phlebotomist at Western University Patient Care Center.

#### Ethical approval:

The research related to human use has complied with all the relevant national regulations, institutional policies and in accordance with the tenets of the Helsinki Declaration, and has been approved by both the Institutional Review Board (IRB) and the Institutional Biosafety Committee (IBC) of Western University of Health Sciences.

#### Informed consent:

Informed consent has been obtained from all individuals included in this study.

### Bacteria Preparation for Infection

The Erdman strain of *M. tb*, which was gifted by Selvakumar Subbian, Rutgers New Jersey Medical School, was used for the infection of isolated human PBMCs. The HN878 and CDC1551 strains were used to infect human THP-1 macrophages as reported earlier (29). All *M. tb* strains were cultured in Middlebrook 7H9 medium (BD and company, Sparks, MD, USA) with 10% albumin-dextrose complex (ADC) at 37°C. After *M. tb* growth reached a logarithmic phase (determined by an optical density (OD) between 0.5-0.8) the bacteria were processed for infection. *M. tb* was centrifuged and resuspended in sterile 1X phosphate-buffered saline (PBS). Sterile 3-mm glass beads (Corning, NY, USA) were added to the PBS buffer and bacteria pellet. The mixture was then vortexed to disperse the clumps. The processed bacteria were then filtered using a 5 μm diameter syringe filter (Corning, NY, USA) to remove large clumps. The filtered bacteria were serially diluted and plated on 7H11 agar plates to determine the concentration of the stocks. The processed bacteria were aliquoted in Cryo Eppendorf tubes and stored in the freezer at −80°C for further use. All *M. tb* handling was carried out inside a certified biosafety level 3 facility under a biosafety cabinet.

### PBMC Isolation, Treatment, Infection and Granuloma Formation

20 mL whole blood samples were layered on top of the same amount of Ficoll-Histopaque reagent (Sigma-Aldrich, St. Louis, MO, USA) in two 50 mL conical centrifuge tubes. After centrifugation at 1800 rpm for 30 mins, the PBMC layer was gently isolated into new 50 mL conical tubes. The isolated PBMCs were washed twice with sterile 1X PBS buffer and suspended in Rosewell Park Memorial Institute (RPMI) medium (Sigma-Aldrich, St. Louis, MO, USA) containing 5% human AB serum (Sigma-Aldrich, St. Louis, MO, USA). The PBMC numbers were determined by Trypan blue (Sigma-Aldrich, St. Louis, MO, USA) exclusion staining with the aid of a hemocytometer.

PBMCs were distributed into 24-well cell culture plates (Corning, NY, USA) coated with 0.001% polylysine overnight at a density of 6x10^5^/well. The *M. tb* Erdman strain was added to the wells at a multiplicity of infection (MOI) of 0.1:1. The infected PBMCs were either sham-treated (control) or treated with 1nM everolimus (LKT Laboratories, St Paul, MN). Based on our extensive experience in generating human *in vitro* granulomas, it is most common to observe solid aggregation of PBMCs to form granulomas approximately 7 days post-infection with *M. tb* [8,21-23]. Therefore, infected PBMCs were terminated at 8- and 15-days post-infection.

### THP-1 Cell Culture, Treatment, and Infection

THP-1 cells were recovered from frozen stock, and DMSO-Cryopreservative medium (Sigma-Aldrich, St. Louis, MO, USA) was removed by washing in 1X sterile PBS. The cells were centrifuged at 2000 rpm at room temperature for 15 min, then resuspended in the RPMI medium with 10% fetal bovine serum (Sigma-Aldrich, St. Louis, MO, USA). The cells were maintained in tissue culture flasks at 37°C, in a 5% CO_2_ incubator. For experimental assays, THP-1 cells were processed as follows: cells were recovered from the flasks and centrifuged at 2000 rpm for 15 min at room temperature. The cell pellet was dispersed and was resuspended in a fresh RPMI medium with 10% fetal bovine serum. The cell numbers of the THP-1 resuspension were determined by Trypan blue. 2X10^5^ cells/well were distributed in 24-well cell culture plates coated with 0.001% poly-lysine. THP-1 macrophages were differentiated by incubation with 10ng/mL phorbol 12-myristate 13-acetate (PMA) (Sigma-Aldrich, St. Louis, MO, USA) overnight at 37°C in a 5% CO_2_ incubator. The media was replaced the following day. Differentiated THP-1 macrophages were infected with clinical *M. tb* isolates HN878 or CDC1551 at an MOI of 5:1 and incubated for 3 hours at 37°C in a 5% CO_2_ incubator for internalization. Following the infection, non-phagocytosed bacteria were removed by washing with warm sterile 1X PBS. Fresh RPMI medium with 10% fetal bovine serum with or without everolimus (0.5ug/ml) was added to the infected THP-1 macrophages. Two extra wells per category were used for Oil red staining. The infected THP-1 macrophages were maintained for 24h, 48h, and 72h post-infection.

### Termination of *in vitro* Granulomas and THP-1 Macrophages for CFUs Assay

Following the different incubation times, the granulomas were terminated on day 8 and day 15 post-infection, and the THP-1 macrophages were terminated on 24h, 48h, and 72h post-infection. The terminated samples were used in the colony-forming units (CFUs) assay to determine the survival of *M. tb* inside the granulomas and THP-1 macrophages. The supernatants of each group were collected into Eppendorf tubes, and 250 μl of ice-cold sterile 1X PBS was added into the cells, followed by scraping of the wells.

### CFUs Assay

The supernatant and lysate samples collected from the termination were vortexed and subjected to three freeze-thaw cycles for the bacteria to fully release from the cells. The samples from different treatment categories were plated on 7H11 agar plates containing albumin dextrose complex (ADC). The plates were incubated for 4-6 weeks until *M. tb* colonies appear and are ready to count.

### Lipid Body Content Measurements by Oil Red Staining in THP-1 Macrophages and Immunofluorescent Staining

For fluorescence staining, macrophages were collected in PBS, and the lipid bodies were stained with Oil red (Sigma-Aldrich, St. Louis, MO, USA) at 0.1 μg/ml, from a stock solution in methanol for 15 min. The samples were then washed with PBS, fixed for 30 min in PBS-PFA 4%, and mounted with the fluorescent DAPI-mounting medium (Thermo Fisher Scientific, Waltham, MA, USA). The slides were analyzed on an EVOS FL cell imaging system (Thermo Fisher Scientific, CA, USA).

### Statistical Analysis

Experimental data were analyzed by using GraphPad Prism Software 8.0. Unpaired t-test with Welch correction was applied for two-sample comparisons. A one-way ANOVA was used for multiple samples comparison with Tukey corrections for the analysis of variance. All the values reported are the means values with each category. A p<0.05 was considered significant and labeled with one asterisk (*). The p<0.005 was labeled with two asterisks (**). The sample size of the pre-clinical trial was eight T2DM positive subjects. The THP-1 study was repeated in six samples.

## Results and Discussion

### Everolimus Restricts Growth of *M. tb* within *in vitro* Human Granulomas from Individuals with T2DM.

**Figure 1:**
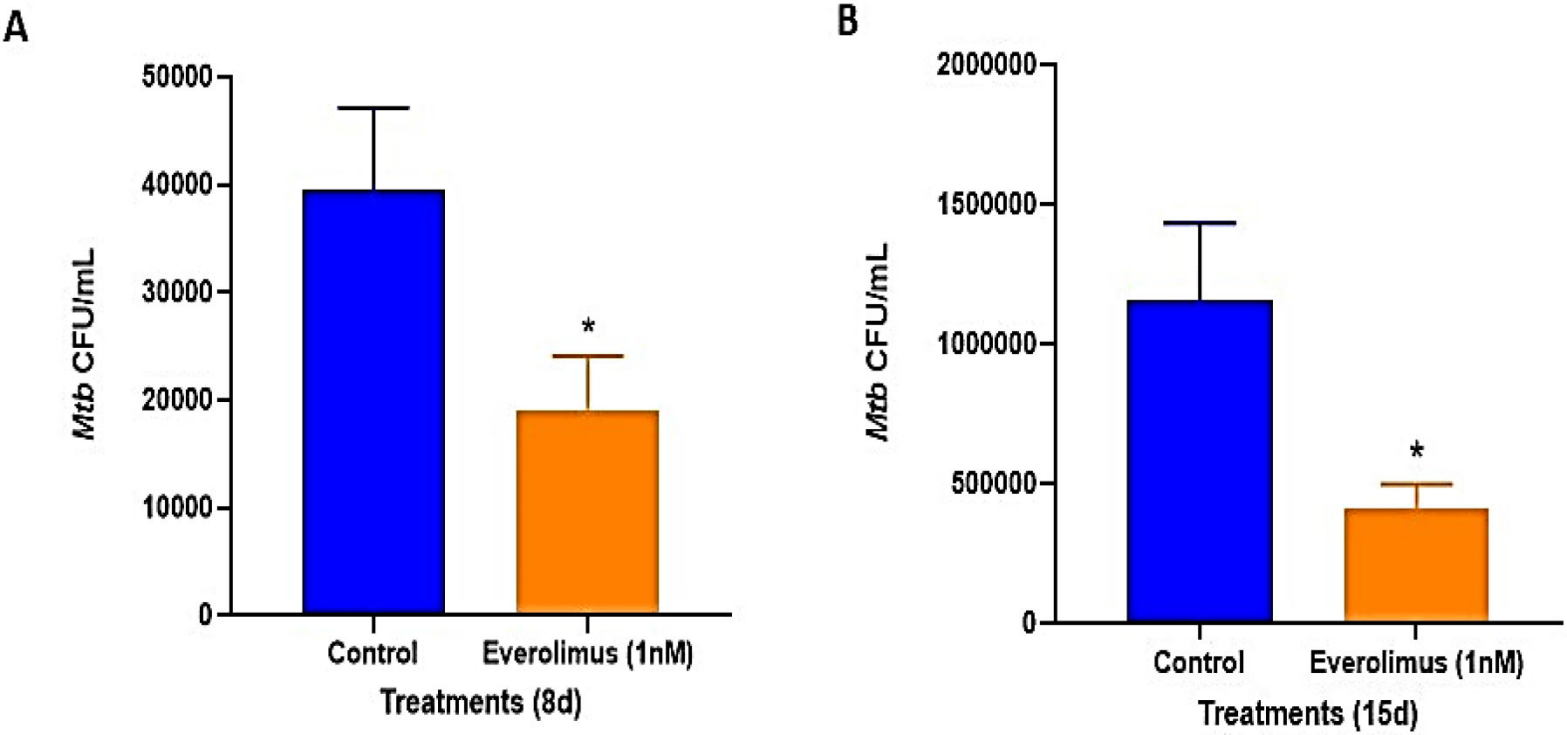
Survival of *M.tb*. Erdman strain in untreated (control) and Everolimus-treated *in vitro* granulomas generated from PBMCs isolated from individuals with type 2 diabetes. PBMCs isolated from individuals with type 2 diabetes were infected *in vitro* with *M.tb*. Erdman strain and were either untreated (control) or treated *in vitro* with everolimus (1 nM). Granulomas were terminated at 8-days (Figure 1A) and 15-days (Figure 1B) post-infection. Cell-free supernatants were collected and stored. Granulomas were lysed with ice-cold PBS. Supernatants and granuloma lysates were plated on 7H11 agar plates containing ADC to determine the survival of *M.tb*.

We first tested the ability of everolimus in inhibiting the growth of intracellular *M. tb* within *in vitro* granulomas generated with PBMCs from individuals with T2DM. The *in vitro* granulomas were either sham-treated or treated with 1 nM everolimus. Granulomas were terminated at 8-days (Figure 1A) and 15-days (Figure 1B) post-infection. There was a significant decrease in bacterial CFUs at both 8 days and 15 days post-infection in granulomas treated with 1 nM everolimus, compared to untreated control groups. These results suggest the efficacy of everolimus against *M. tb* infection within *in vitro* granulomas generated with PBMCs from individuals with uncontrolled T2DM.

Our previous study in healthy subjects suggests that the elemental mechanism of everolimus in controlling *M. tb* infection is due to its attributes in augmenting autophagy, decreasing ROS production, and reducing the production of proinflammatory cytokines, therefore decreasing *M. tb* burden within *in vitro* granulomas [8]. The control of intracellular survival of *M. tb* is a complex process not only affected by the host cell but by the bacillus itself as well. Despite the mechanisms we explored, much is still poorly understood.

During *M. tb* infection, the granuloma becomes highly vascularized and recruits lymphocytes, macrophages, and dendritic cells to fight the infection. A special group of cell types found within the granuloma structure during *M. tb* infection are the foamy macrophages. These cells play a large role in either localized *M. tb* infection and mineralization of the lesion or the process of caseation and necrosis that further evokes the reactivation of *M. tb*. The main characteristic of foamy macrophages is the overexpression of lipid bodies. As the granuloma matures and macrophages differentiate, foamy macrophages begin to develop due to an uneven amount of LDL entering and exiting the cell [15]. Peyron et al. have demonstrated that *M. tb* long-chain fatty acids (oxygenated mycolic acids) can also trigger the differentiation of macrophages into foam cells [18]. Foamy macrophages are not only the product of an inflammatory response, but they are also inflammatory cells themselves. Our recent research has shown that treatment with everolimus can downregulate the oxidative stress and TNF-α levels (which elevate with inflammation) and attenuate the inflammatory responses within human *in vitro* granulomas [8]. Therefore, it is crucial to test the effects of everolimus treatment in altering the hemostasis of foamy macrophages (such as lipid body levels) and link the intracellular survival of *M. tb* within foamy macrophages to the lipid components load.

### Everolimus Reduces the Intracellular Survival of *M. tb* HN878 and CDC1551 in THP-1 Macrophages

**Figure 2:**
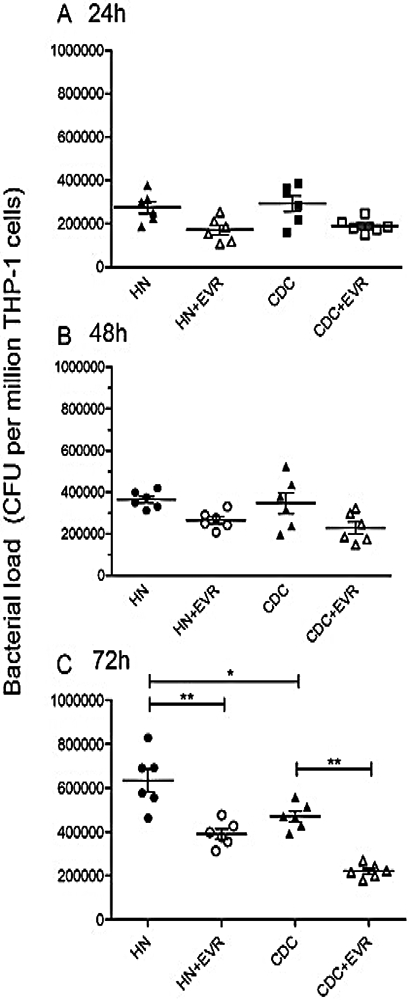
Survival of *M.tb* in THP-1 cells. THP-1 cells were infected *in vitro* with CDC 1551 and HN878 strains of *M.tb*. Infected THP-1 cells were either untreated (control) or treated *in vitro* with everolimus (0.5 mg/ml). Infected macrophages were terminated at 24h (Figure 2A), 48h (Figure 2B) and 72h (Figure 2C) post-infection. Cell-free supernatants were collected and stored. Granulomas were lysed with ice-cold PBS. Supernatants and granuloma lysates were plated on 7H11 agar plates containing ADC to determine the survival of *M.tb*.

We induced a foamy macrophage model using THP-1 macrophages and evaluated the effects of everolimus on *M. tb* HN878 or CDC1551 infected THP-1 macrophages. We determined the intracellular survival of *M. tb* in HN878 or CDC1551 infected THP-1 macrophages by CFU assay. To illustrate the antimicrobial ability of everolimus inside macrophages, THP-1 macrophages were infected by either HN878 or CDC1551 at MOI 5:1, the viability of *M. tb* was determined by CFU assay at 24 hrs, 48 hrs, and 72 hrs post-infection time points. Consistent with our findings in human *in vitro* granulomas studies, everolimus decreased the viability of *M. tb* in the THP-1 macrophages at the 72 hrs post-infection timepoint. However, there were no significant differences in bacterial killing at the 24 hrs and 48 hrs post-infection time points (Figure 2A, B, C). These observations suggest that foamy macrophages phenotype is established during later time of infection (72hrs) and everolimus treatment is more pronounced only when the macrophages are in a foamy phenotype. Thus everolimus treatment of foamy macrophages can restrict bacterial proliferation, although it does not kill all the bacilli during early time points (Figure 2A, B, C).

The underlying mechanisms of everolimus in the killing of *M. tb* within foamy macrophages need to be investigated. The foamy macrophages are known for rich-lipid body contents and act as a pivotal nutrient resource for bacilli persistence. We speculated that the enriched lipid bodies may cause the disability of foamy macrophages in inhibiting the intracellular *M. tb* growth, and everolimus may play a role in preferentially scavenging the lipid bodies inside the foamy macrophages.

### *M. tb* Infection Causes an Increase in Lipid Body Levels within THP-1 Macrophages

Next we tested the effect of Mtb infection in promoting a foamy phenotype to infected macrophages. We found that the infection of THP-1 macrophages by both HN878 and CDC1551 strains caused a significant increase in lipid body contents within the THP-1 macrophages, compared to uninfected groups in all three post-infection timepoints (24hrs, 48hrs, and 72 hrs), which also correlates with a higher CFU burden. Our data support the hypothesis that the formation of lipid bodies impacts the bactericidal ability of foamy macrophages.

It has been demonstrated that foamy macrophages exist in leprosy patients, *Mycobacterium avium* infected HIV patients, and chronic *M. tb* infected mice [24-26]. As a result, lipid body contents accumulate in those conditions. Frederic Altare’s laboratory has tested multiple bacterial strains of*M. tb, M. smegmatis*, and *M. avium* and found that they all triggered foamy macrophage formation [18]. They suggested that macrophages lost the capability to control the growth of *M. tb* once they were induced into foamy macrophages [18]. Our study also demonstrates that the formation of lipid bodies has a negative correlation with intracellular *M. tb* control. Several hypotheses explain how foamy macrophages inhibit *M. tb* clearance. Russel et al. suggest that the conversion of macrophages into foam cells is a pathogen-driven process that eventually can lead to reactivation of the bacteria and death of the macrophages [27]. Therefore, manipulating the host tissue is a promising therapy. This is supported by the work of Pandey and Sassetti, who show that *M. tb* can metabolize cholesterol to survive in the foamy macrophages, characterizing the transformed macrophages as beneficial to the bacteria rather than detrimental [28].

### Everolimus Reduces Lipid Body Contents within THP-1 Macrophages Infected by CDC1551 and HN878

Everolimus has been proposed to be a promising host-directed therapy in conjunction with front-line antibiotics in treating TB. In our previous study, we have tested the effects of everolimus in the downregulation of the oxidative stress levels, elevating autophagy of human in vitro granulomas, and inhibiting the intracellular growth of *M. tb* [8]. However, the correlation between the levels of lipid body contents within the immune cells and the ability to restrict *M. tb* replication within the host cells has been untapped. Based on our preliminary findings, we postulate that everolimus also plays a critical role in the lipid metabolism of host cells and the fate of*M. tb* infection. We administered 0.5 μg/mL everolimus to differentiated THP-1 macrophages infected with either CDC1551 or HN878. Treatment with everolimus significantly decreased the levels of lipid bodies inside the THP-1 macrophages at all time points (24hrs, 48hrs, and 72hrs post-infection) when compared with sham-treated groups. This supports our hypothesis that everolimus can downregulate the lipid components caused by the *M. tb* infection in the THP-1 macrophages, indicative that diminished lipid bodies can therefore decrease the viability of *M. tb*.

## Conclusion

Our work unfolds a novel underlying mechanism of action by which everolimus-treated macrophages control *M. tb* infection. Although everolimus may inhibit the growth of *M. tb* in several ways, findings from this study indicate that reduction in the lipid body content of infected macrophages may play a significant role in enhancing the antimycobacterial defense. In this study, THP-1 macrophages infected with *M. tb* were shown to have elevated levels of lipid bodies, and THP-1 macrophages treated with everolimus were shown to have decreased levels of lipid bodies. Concurrently, *M. tb*-infected THP-1 macrophages treated with everolimus were shown to have a reduced level of *M. tb* survival. These results provide further evidence in favor of using everolimus as a possible adjunctive therapy for TB.

## Figures and Tables

**Figure 3:**
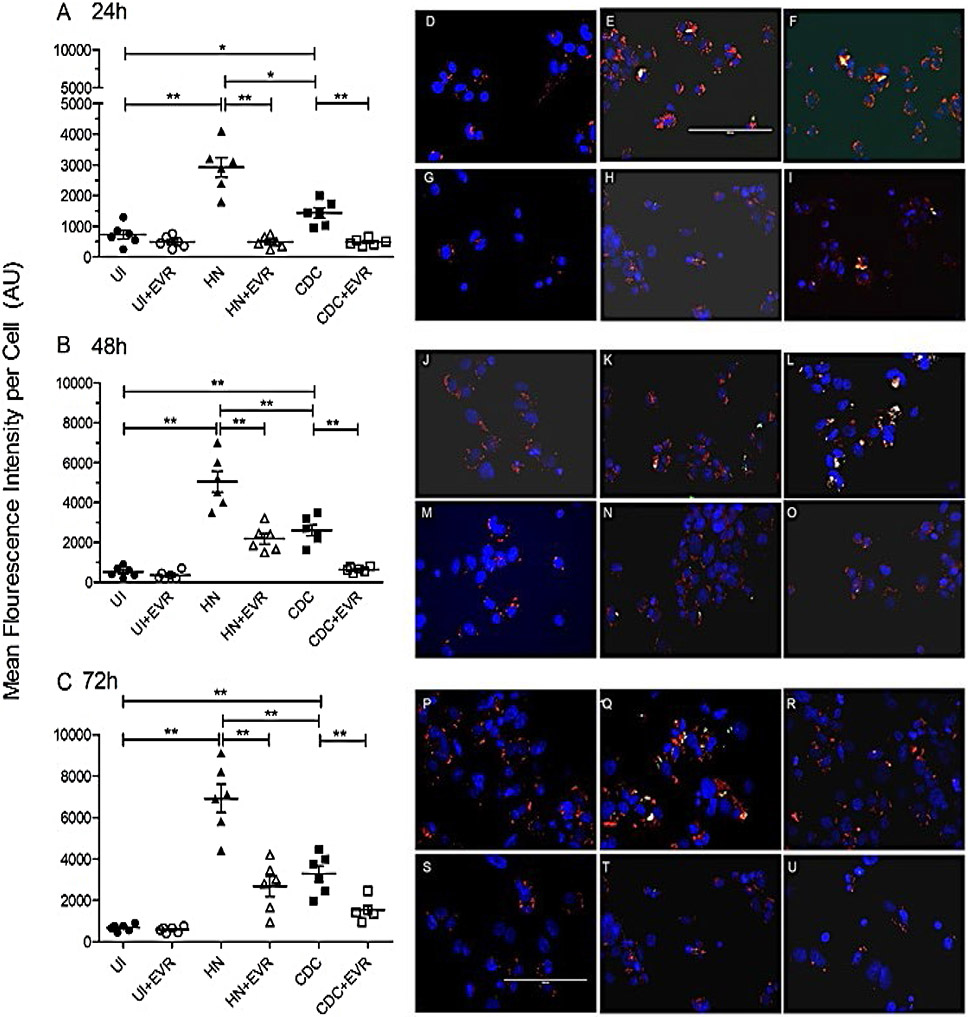
Quantification of Mean Fluorescent Intensity for Oil Red Staining: *M. tb* (CDC1551 or HN878)-infected macrophages cultured in the presence and absence of everolimus (0.5 mg/ml) were terminated at 24h (Figure 3A), 48h (Figure 3B), and 72h (Figure 3C) post-infection. Lipid bodies were stained with Oil red (Sigma-Aldrich, 0.1 μg/ml, from a stock solution in methanol) for 15 min. The samples were then washed with PBS, fixed for 30 min in PBS-PFA 4%, and mounted with the fluorescent DAPI-mounting medium. The slides were observed under a confocal microscope. Mean Fluorescent Intensity was calculated using ImageJ software. Figure 3D, E, F, G, H, I, J, K, L, M, N, O, P, Q, R, S, T and U are images showing Oil Red Staining. *M. tb* (CDC or HN878)-infected macrophages cultured in the absence (Figure 3D, E, F, J, K, L, P, Q, R) and presence (Figure 3G, H, I, M, N, O, S,T,U ) of everolimus (0.5 mg/ml) were terminated at 24h (D,E,F,G,H,I), 48h (J,K,L,M,N,O) and 72 h (P, Q,R,S,T,U) post-infection. Lipid bodies were stained with Oil red for 15 min. The samples were then washed with PBS, fixed for 30 min in PBS-PFA 4%, and mounted with the fluorescent DAPI-mounting medium. The slides were observed under a confocal microscope. Images were captured under 40X.

## Data Availability

The datasets generated during and/or analysed during the current study are available from the corresponding author on reasonable request.
